# Physiologically Based Biopharmaceutics Model (PBBM) of Minimally Absorbed Locally Acting Drugs in the Gastrointestinal Tract—Case Study: Tenapanor

**DOI:** 10.3390/pharmaceutics15122726

**Published:** 2023-12-04

**Authors:** Konstantinos Stamatopoulos, Nena Mistry, Nikoletta Fotaki, David B. Turner, Brandon Swift

**Affiliations:** 1 Biopharmaceutics, DPD, MDS, GSK, Ware SG12 0DP, UK; nena.2.mistry@gsk.com; 2 Centre for Therapeutic Innovation, Department of Life Sciences, University of Bath, Claverton Down, Bath BA2 7AY, UK; nf223@bath.ac.uk; 3 Certara UK Limited, Simcyp Division, Sheffield S1 2BJ, UK; david.turner@certara.com; 4 GSK, Durham, NC 27709, USA; brandon.x.swift@gsk.com

**Keywords:** tenapanor, minimally absorbed, food effect, PBBM, IBS, NHE3, GI tract, locally acting drugs, biopharmaceutics

## Abstract

A physiologically based biopharmaceutics model (PBBM) was developed to predict stool and urine sodium content in response to tenapanor administration in healthy subjects. Tenapanor is a minimally absorbed small molecule that inhibits the sodium/hydrogen isoform 3 exchanger (NHE3). It is used to treat irritable bowel syndrome with constipation (IBS-C). Its mode of action in the gastrointestinal tract reduces the uptake of sodium, resulting in an increase in water secretion in the intestinal lumen and accelerating intestinal transit time. The strategy employed was to perform drug–drug interaction (DDI) modelling between sodium and tenapanor, with sodium as the “victim” administered as part of daily food intake and tenapanor as the “perpetrator” altering sodium absorption. Food effect was modelled, including meal-induced NHE3 activity using sodium as an inducer by normalising the induction kinetics of butyrate to sodium equivalents. The presented model successfully predicted both urine and stool sodium content in response to tenapanor dosed in healthy subjects (within 1.25-fold error) and provided insight into the clinical observations of tenapanor dosing time relative to meal ingestion. The PBBM model was applied retrospectively to assess the impact of different forms of tenapanor (free base vs. HCl salt) on its pharmacodynamic (PD) effect. The developed modelling strategy can be effectively adopted to increase confidence in using PBBM models for the prediction of the in vivo behaviour of minimally absorbed, locally acting drugs in the gastrointestinal tract, when other approaches (e.g., biomarkers or PD data) are not available.

## 1. Introduction

The study of minimally absorbed locally acting drugs (LADs) poses unique challenges in comparison to drugs with measurable systemic concentrations which can be utilised to develop physiologically based pharmacokinetic (PBPK) models to potentially address biopharmaceutic and drug–drug interaction questions. LADs are not intended to be absorbed into the bloodstream, a fact which reduces the available data to validate and accurately predict drug–drug interactions [1].

To obtain approval for a new LAD drug, demonstration of clinical efficacy and availability of safety data are crucial, while comparative clinical endpoint bioequivalence (BE) studies may be required for post pivotal changes to the solid oral dosage form. However, these comparative clinical studies can be costly and may not be sensitive enough to detect differences in formulation when the exposure–response relationship is flat.

For minimally absorbed LADs in the GI tract, pharmacodynamic (PD) data, such as biomarkers and clinical outcomes, are currently the primary means of developing a drug that is safe and efficacious and demonstrating BE, unless a waiver is possible per local regulatory guidance (e.g., highly soluble immediate release drug products).

With regard to PBPK modelling for these types of drugs, the clinical endpoints might be measured over an extended period (weeks or months), depending on the response time frame compared to the dose schedule, making it difficult to use these data to develop and validate a PBPK model to understand regional drug concentrations in the GI tract.

In certain cases, PD data may potentially be linked to the gastrointestinal distribution of LADs. Minimally absorbed LADs may alter the composition of the gut lumen fluids by blocking the active transport of endogenous or exogenous molecules, such as bile salts or cations like sodium. These changes may be reflected in the composition of faeces as a function of dose [2,3] and meal-to-dosing intervals [4]. Additionally, these changes in luminal composition may alter intestinal fluid secretions and motility in the GI tract as a response to the disruption of the lumen physiology. In particular, Rosenbaum et al. [2] showed that the weight of the faeces, water content, and bowel movements increased when the absorption of sodium in the gut was blocked by tenapanor, a sodium/hydrogen isoform 3 exchanger (NHE3) inhibitor with minimal systemic exposure [3,5].

In the gastrointestinal tract, sodium is released by a meal, upon digestion, into the lumen and it is actively absorbed by NHE3, [6] localised on the apical membrane of enterocytes [7]. The activity of NHE3 at the apical membrane of epithelial cells is highly regulated by a number of mechanisms, including transcription, protein phosphorylation, protein–protein interaction, and trafficking [8]. NHE3 is a unique sodium/hydrogen exchanger in that modulation of activity can occur acutely via a rapid, reversible recycling between the apical membrane and intracellular endosomes that occurs within minutes to hours [9]. This acute regulation is linked to the biological role of NHE3 in intestinal sodium and water homeostasis [10]. Maher et al. [11] showed that the water and ion absorption rate increased in the small intestine approximately 30 min after meal ingestion in canines, while Pasham et al. [12] demonstrated significant upregulation of intestinal NHE3 following saline ingestion in mice. In addition, induction of NHE3 by short chain fatty acids (SCFA), a food digestion byproduct, have been reported in rat colon and human intestinal C2/bbe cells, in which NHE3 expression increased within 6h and reached steady state after 12h when incubated with SCFA [13].

Considering this rapid response to meal-stimulated NHE3 activation as well as the inhibitory effect of tenapanor, physiologically based biopharmaceutics modelling (PBBM) can be used to simulate this dynamic interplay between meal-stimulated activity and inhibition of NHE3 to elucidate the impact on sodium absorption. For this purpose, PBBM of tenapanor was developed to describe the regional distribution of tenapanor in the gastrointestinal tract, and was validated indirectly by predicting the amount of sodium in the faeces of healthy human volunteers due to changes in its absorption. Sodium is a dietary component, administered to humans with food, whose presence stimulates NHE3 activity as mentioned earlier. To model sodium intake by diet, the amount contained in the food is used as the “dose” administered with or without tenapanor. The ability of the model to describe the observed food effect on tenapanor-mediated NHE3 inhibition was also explored. Finally, the opportunities and challenges associated with PBBM of minimally absorbed LADs are discussed.

## 2. Methods

### 2.1. PBBM Development

**Figure 1:**
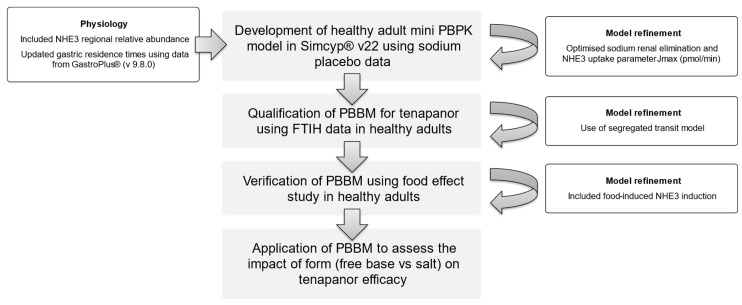
Workflow for PBBM development, qualification, verification, and application.

Simcyp^®^ v22 (Certara division, Sheffield, UK) was used to run the simulations in a healthy human population. The Gastroplus^®^ (v 9.8.0) (Simulation Plus, Lancaster, CA, USA) database was used for gastric residence times to update the Simcyp^®^ population table. The mechanistic ADAM model was used to handle dissolution using the Diffusion Layer Model (DLM), as described by Sugano [14]. The Simcyp In Vitro Data Analysis (SIVA v4-Certara division, Sheffield, UK) tool was used to parameterise the ADAM model. Figure 1 shows the workflow followed to develop the PBBM, and Table 1 details the model input parameters.

### 2.2. Sodium, Tenapanor, and Food Effect Modelling

To model food–tenapanor interactions impacting Na+ absorption, Na+ was set as the “substrate” dosed per food consumption. In terms of solubility and dissolution, sodium was assumed to be in solution and available for absorption. As Na+ is actively absorbed in the human gut [19], passive permeability was set to zero. Active transport of Na+ by NHE3 was simulated using the apical influx (intestine) option in Simcyp^®^, and relative regional abundance and enzyme kinetics were set. Sodium renal elimination was optimised to capture the urine Na+ content after the administration of the placebo.

The amount of sodium parameterised in the simulations was based on a standardised diet consisting of three daily meals that contained approximately 1.1–1.5 g (48–65 mmol) of sodium, equivalent to 8.4–11.4 g of table salt per day [2].

Tenapanor is a BCS class IV drug [15] with negligible systemic exposure [20]. It is presented as a hydrochloride (HCl) salt immediate release (IR) formulation. SIVA v4 was used to estimate the intrinsic solubility of the free base to inform the salt model (Figure S1). Tenapanor is a highly protein-bound drug (>98%), and the maximum observed free drug concentration in human plasma (<0.015 nM) is well below its in vitro inhibitory potency (IC_50_:5 nM) [3]. Thus, tenapanor is not expected to inhibit NHE3 in the kidney due to minimal systemic exposure, and, therefore, we did not account for inhibition of NHE3 in the kidney compartment.

The minimum PBPK model in Simcyp was used to estimate the volume of the distribution using Method 2 for sodium and tenapanor.

Food-stimulated NHE3 activity was simulated using regional sodium intestinal concentrations. Previously published butyrate data after normalisation to Na+ equivalents were used to estimate food-stimulated NHE3 activity kinetic parameters [14].

### 2.3. Clinical Studies

The following clinical studies were used for model development, qualification, and verification:

Model development and qualification:

Rosenbaum et al. [2]—participants: healthy volunteers. Dosing schedule: multiple ascending doses (placebo, 15 mg b.i.d (twice daily), 30 mg b.i.d, 30 t.i.d (three times daily), 60 mg b.i.d and 30 mg q.d (once daily)). Analysis: the urine and stool sodium contents were reported as mmol/day with samples taken every 24 h (post dose/placebo) per subject for 9 days. Dosing started on day 2 and ended on day 8.

Spencer et al. [3]—clinical design: once daily, doses of tenapanor of increasing strength (3 to 100 mg) were given to healthy volunteers (*n* = 6 per group), and the average daily faecal sodium was determined. A correlation between stool and urine sodium content in response to tenapanor (30 mg q.d, b.i.d, and t.i.d, 15 mg b.i.d and 60 b.i.d daily for 7 days) in healthy subjects was reported as the difference with respect to the placebo-induced sodium content based on the cumulative amount of sodium (Na+) excretion on day 7.

Model verification:

Johansson et al. [4]—clinical data reported as average (95% confidence interval) daily stool sodium excretion (mmol/day) over 4 days of treatment with tenapanor administration (15 mg IR tablets b.i.d) 5–10 min before a meal (breakfast and dinner), 30 after a meal (breakfast and dinner), and fasting 1 h before breakfast and 3 h after dinner.

### 2.4. Statistical Analysis

The average absolute fold error (AAFE) was calculated, as described elsewhere [21,22,23], to assess the overall predictive accuracy of the model. The average fold error (AFE) was calculated, as described elsewhere [24], to assess the tendency for over- or underprediction of the model as well as predicted/observed ratio.

## 3. Results

### 3.1. Model Qulaification

**Figure 2:**
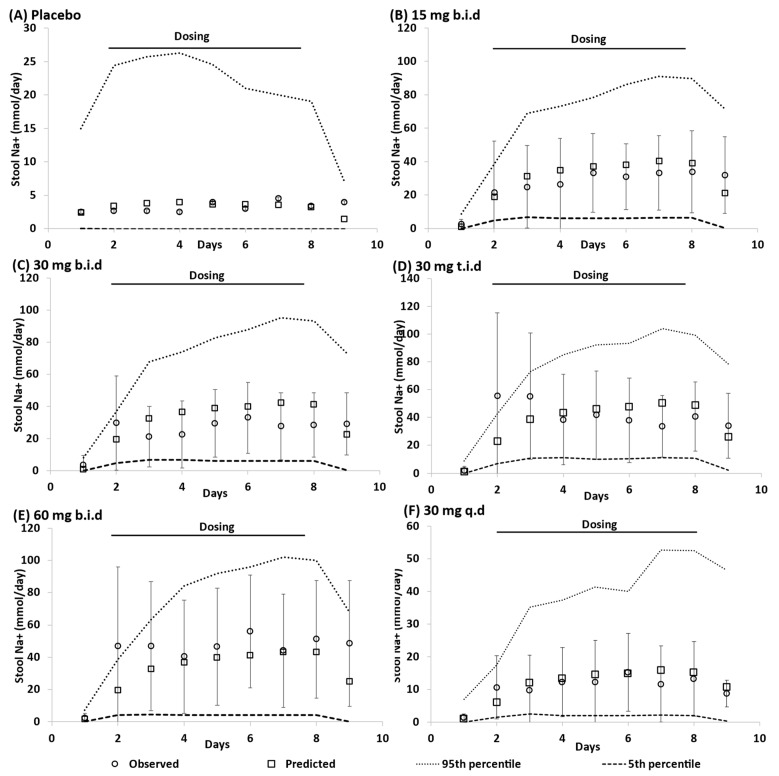
Daily faecal excretion of sodium (Na+) in healthy volunteers treated with tenapanor or placebo [2]. Placebo (**A**); 15 mg b.i.d. (**B**); 30 mg b.i.d. (**C**); 30 mg t.i.d (**D**); 60 mg b.i.d. (**E**); 30 mg q.d (**F**). b.i.d. twice daily, q.d. once daily, t.i.d. three times daily. Observed data (○ mean ± SD); the horizontal solid line represents the start (Day 2) and end (Day 8) of tenapanor dosing.

The PBBM developed was qualified by assessing its predictive accuracy against daily Na+ excretion (mmol) in faeces and urine (Figure 2) after the administration of tenapanor with different dosing schedules.

**Figure 3:**
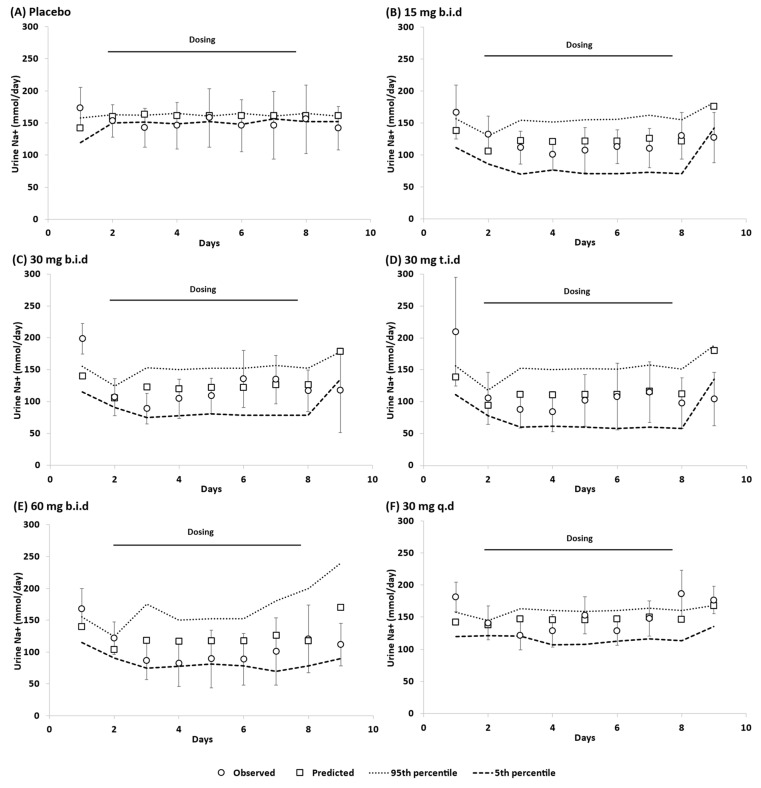
Daily urinary excretion of sodium (Na+) in healthy volunteers treated with tenapanor or placebo [2]. Placebo (**A**); 15 mg b.i.d. (**B**); 30 mg b.i.d. (**C**); 30 mg t.i.d (**D**); 60 mg b.i.d. (**E**); 30 mg q.d (**F**). b.i.d. twice daily, q.d. once daily, t.i.d. three times daily. Observed data (mean ± SD; open circles); the horizontal solid line shows the start (Day 2) and end (Day 8) of the dosing schedule for tenapanor.

Model simulations of daily Na+ excretion in faeces (Figure 2) and urine (Figure 3) before and after tenapanor administration with different dosing schedules and strengths were in good agreement with the clinical data reported by Rosenbaum et al. [2].

### 3.2. Food Effect

**Figure 4:**
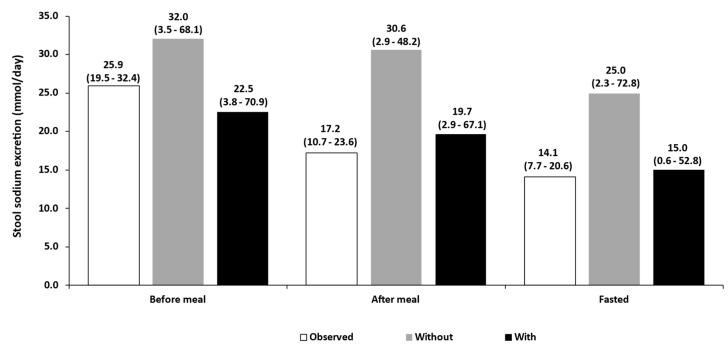
Predicted versus observed average (95% confidence interval) daily sodium excretion (mmol/day) in stool over 4 days of treatment with tenapanor administration (15 mg IR tablets twice daily). Before meal (breakfast and dinner): 5–10 min; After meal (breakfast and dinner): 30 min; Fasting: 1 h before breakfast and 3 h after dinner [4] with and without induction of NHE3 upon food ingestion.

Figure 4 shows the predicted versus observed average daily sodium excretion (mmol) in stool when tenapanor (15 mg IR tablets twice daily) is administered at different times relative to meal intake [4], and considering induction of NHE3. The results show that when tenapanor is administered 5–10 min before a meal, induction of NHE3 does not significantly affect the predictions, although the performance of the model experienced a slight decrease following induction while remaining within an acceptable 1.25-fold error threshold. This was likely due to overestimation of the induction of NHE3, leading to increased enterocyte uptake of sodium resulting in lower excretion in faeces. However, the importance of including induction can be seen when tenapanor was administered after a meal or in fasted state. Without including NHE3 induction, the model overestimated the inhibition of NHE3 by tenapanor, leading to overprediction of sodium excretion in faeces compared to the observed clinical data.

**Figure 5:**
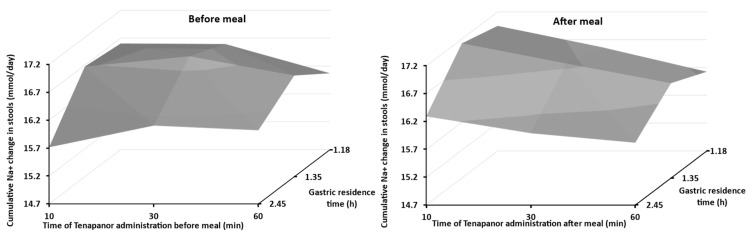
Impact of gastric residence times and drug administration (15 mg twice daily for 4 days) relative to mealtime on the daily stool sodium (Na+) content.

Due to practical and ethical reasons, the participants in the tenapanor food effect studies received “normal” meals rather than high-fat, high-calorie meals (as recommended by FDA guidance) because the study design evaluates the effect over the course of four days [4]. Thus, the model (including NHE3 induction) was used to assess how different meal types might affect tenapanor-mediated NHE3 inhibition by examining changes in sodium excretion in stools. Meal-specific gastric residence times (GRTs) were added based on the values used in Gastroplus (v 9.8.0), as these are not provided in Simcyp. The values were used to assess the impact of low, moderate, and high-fat/calorie meals on the Na+ excretion in stool. Figure 5 shows the impact of gastric residence time and tenapanor administration relative to mealtime on the cumulative Na+ change in stool (mmol). As expected, the maximum efficacy of tenapanor was achieved when administered before a meal, regardless of the meal type. A slight reduction in the predicted cumulative amount of sodium excreted was found when tenapanor was administered 10 min before a high-fat/calorie meal (2.45 h gastric residence time). However, this reduction was minimal (6%) and likely not clinically relevant.

The model predicted lower excretion of Na+ in stools (peak value of 75.8 mmol after meal vs. 77 mmol before meal) when tenapanor was administered after meal, although without attaining statistical significance. This was expected, as the Na+ emptied from the stomach would be absorbed before tenapanor and would be able to inhibit NHE3. The tendency, observed in this work, to reduce the efficacy of tenapanor when it was administered after a meal was in accordance with the clinical observations [2,3,4].

However, following examination of the predictions, the model was found to have low sensitivity to changes in gastric emptying and to the time of tenapanor administration before or after a meal. The ratio of Na+ change in stool (mmol/day), in Figure 5, between the best-case scenario (1.35 h GRT with tenapanor administered 10 min before—16.6 mmol) and the worst-case scenario (2.45 h GRT with tenapanor given 10 min after meal—16.3 mmol/day) was only 2%.

The lack of significant difference in cumulative Na+ excretion is likely explained by the predicted luminal concentrations of tenapanor being 1–2 log units above the IC_50_ (i.e., 0.005 μM) in all the GI tract compartments, regardless of the scenario explored in this work (Figure S2). Thus, taking tenapanor before a meal ensures that the luminal concentration of tenapanor will be high enough to inhibit NHE3 regardless of the meal type.

### 3.3. Dose Escalation Study

**Figure 6:**
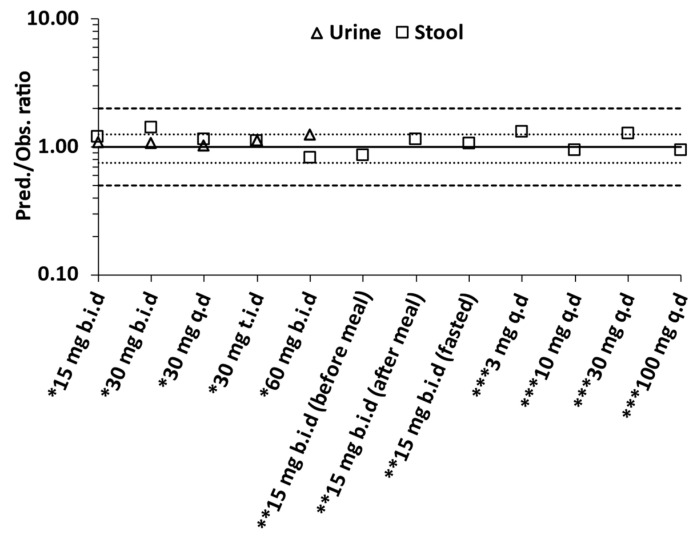
Observed versus predicted ratio of excretion of sodium in urine and stool. The solid line represents the line of unity, the dotted lines represent the 1.25-fold error thresholds, and the dashed lines the 2-fold error thresholds. * Rosenbaum et al. [2]; ** Johansson et al. [4]; *** Spencer et al. [3].

The model was also verified against clinical data of the single ascending dose escalation study (once daily doses of tenapanor of increasing strength (3 to 100 mg q.d) administered to healthy volunteers) [3]. The PBBM was able to capture the average daily faecal excretion of Na+ in fed, healthy volunteers for most of the doses administered (Table 1) within 1.25-fold error (Figure 6: see 3–100 mg q.d data). The model slightly overpredicted the stool sodium content for the 3 mg q.d and 30 mg q.d doses, although it did so within a 2-fold error.

### 3.4. Overall Predictive Accuracy

Table 2 summarises the predicted and observed daily Na+ excretion rate across all three clinical studies used to develop, qualify, and verify the model. The AAFE values for Na+ excretion in urine and stool for observed and simulated values were found to be 1.05 and 1.18, respectively, while the AFE values were 1.11 and 1.10 for sodium excretion in urine and stool, respectively (Table 2).

Figure 6 shows the overall predictiveness of the model by taking the ratio of predicted to observed sodium excretion values in urine and stool. All ratios were within 1.25-fold except for one value at 1.33-fold.

**Figure 7:**
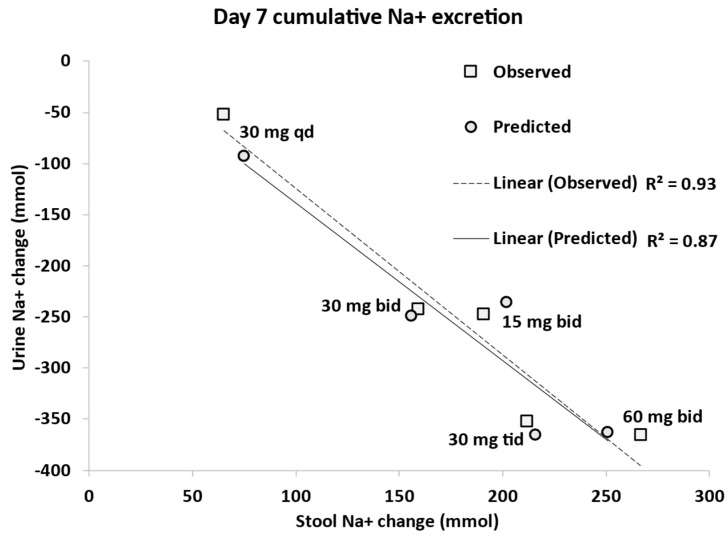
Observed and predicted correlation between stool and urine sodium content in response to different dosing regimens of tenapanor in healthy subjects. Observed data derived from Spencer et al. [3]. The change in urine/stool sodium (Na+) content refers to the cumulative amount of Na+ excretion on day 7 with respect to the placebo.

Figure 7 shows the observed and predicted correlation between stool and urine Na+ content in response to different doses of tenapanor in healthy subjects. The model produced the same strong correlation (R^2^ = 0.87) consistent with the observed data (R^2^ = 0.93 [3]) between the reduction in urinary sodium excretion and the corresponding increase in faecal sodium excretion.

### 3.5. Model Application

**Figure 8:**
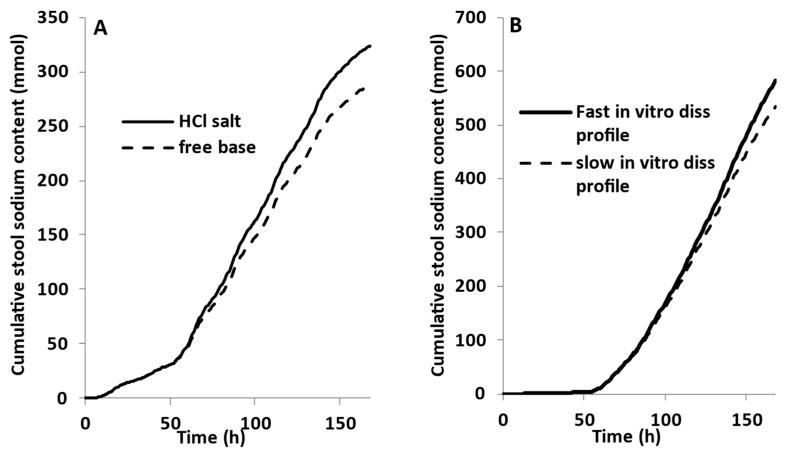
Predicted cumulative stool sodium content (mmol) in response to tenapanor (30 mg b.i.d—7 days—IR tablet) dosed in healthy subjects. (**A**) comparison of HCl salt and free base of tenapanor dosed separately using the mechanistic diffusion layer model (DLM); (**B**) comparison of fast and slow in vitro dissolution profiles as reported in the tenapanor monograph [15].

In the tenapanor monograph, the mixtures of free base and HCl salt forms of tenapanor were used to assess the impact of the dissolution rate of tenapanor in citrate buffer at pH 4 [15]. As expected, in vitro dissolution data showed a higher rate as the percentage of HCl salt was increased in the total (base + salt) combination [15]. The verified PBBM model was used to assess the impact of these changes in the in vitro dissolution rate on the in vivo pharmacodynamics (i.e., Na+ excretion rate) of tenapanor. Thus, the fastest and the slowest dissolution profiles were selected assuming that the fast profile corresponded to the HCl salt and the slow to the free base (the exact base:salt combinations in the monograph are concealed from public disclosure) [15]. The cumulative stool sodium content predicted using the mechanistic DLM or the in vitro dissolution profiles as a direct input to the PBBM are depicted in Figure 8. Using the DLM, the PBBM model predicted a 12% decrease in the cumulative stool sodium content (mmol) between the HCl salt and the free base of tenapanor (30 mg b.i.d tablet—7 days dosing). Using the fast and the slow in vitro dissolution profiles by direct input (Figure 8B), the PBBM predicted a 9% decrease between the two profiles in the cumulative stool sodium content (mmol).

These results demonstrate that the differences observed in the in vitro dissolution profiles do not lead to significant differences in the in vivo performance of tenapanor. The lack of difference in sodium excretion is likely explained by the exceedingly high luminal concentrations of tenapanor, 1-2 fold higher (depending on which in vitro dissolution profile or model was used as an input in the PBBM) than the IC_50_ (0.005 μM).

**Figure 9:**
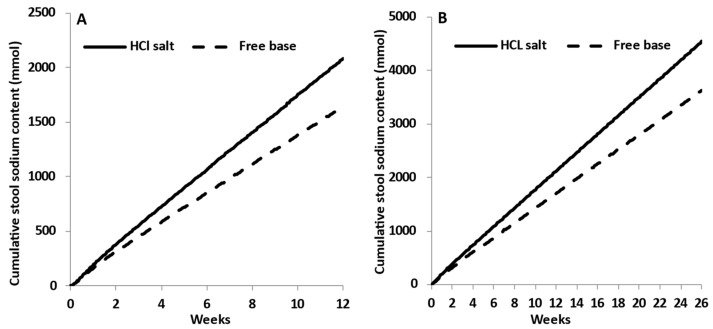
Predicted cumulative stool sodium content (mmol) in response to tenapanor (50 mg b.i.d) administered to healthy subjects. Comparison of HCl salt and free base forms of tenapanor administered over 12 weeks (**A**) and 26 weeks (**B**) using the mechanistic Diffusion Layer Model (DLM).

Tenapanor is used for treatment of irritable bowel syndrome with constipation (IBS-C) in adults. Treatment for IBS-C can be chronic, lasting for months or longer using different lines of treatment [25,26]. Thus, the simulations were extended to 12 weeks [27] and 26 weeks [28] for assessment of the long-term PK of tenapanor 50 mg b.i.d. for the treatment of patients with IBS-C. Figure 9 shows the comparison of the predicted cumulative stool sodium content (mmol) between the tenapanor HCl salt and free base.

Extrapolation of predictions to 12 and 26 weeks showed that the difference between the two forms of tenapanor experienced an increase with the HCl salt, providing higher stool sodium excretion compared to the free base. The mean daily sodium content in stool at steady state was 10.0 and 7.7 mmol/day for the HCl salt and free base, respectively, over 12 weeks of treatment. The corresponding daily stool excretion for the 26-week treatment was 22.3 mmol/day and 17.0 mmol/day for the HCl salt and free base, respectively. This is a 1.3-fold difference between the two forms compared to the 1.05-fold difference based on the predictions in the first week. The predicted difference between the HCl salt and free base with respect to the placebo was 4.2- and 3-fold, respectively.

## 4. Discussion

In the present work, a PBBM was developed to predict stool and urine sodium content before and after various tenapanor dosing regimens in healthy subjects. The model successfully predicted within 1.25-fold error both urine and stool sodium content. The model was also applied to assess the impact of the timing of a meal relative to tenapanor administration and the fat content of the meal on tenapanor-mediated inhibition of Na+ absorption. In addition, the effect of tenapanor dosage was evaluated on the long-term pharmacodynamic effect.

Typically, the concept of pharmacokinetic bioequivalence acts as a stand-in to determine therapeutic equivalence when comparing a generic formulation with an innovator product or two formulations upon pivotal or post-pivotal changes during drug product development. Nonetheless, the concept of pharmacokinetic bioequivalence cannot be applied when dealing with drugs that exhibit low systemic bioavailability below the limit of assay quantification and extremely variable. Thus, many drug products requiring bioequivalence studies have used PD endpoints in lieu of PK parameter comparisons [29]. However, the clinical PD endpoints might be measured over an extended period (weeks or months) depending on the response time frame compared to the dosing schedule, making it difficult to use these data to develop and validate a PBBM model to understand regional drug distribution in the GI tract.

Modelling the regional concentrations of non- or minimally absorbed drugs that target a site of action in the gut wall requires accounting for changes in the GI tract physiology as well as in the luminal environment and how these changes are propagated, resulting in further changes in gastrointestinal behaviour. For instance, tenapanor blocks the absorption of sodium and its accumulation in the colon increases bowel movements and stool weight in a dose-proportional manner [2]. It is challenging to develop a PBBM which accounts for these downstream changes, as there are several underlying mechanisms that act as a ‘domino effect’ and cause alterations to gut motility. For example, high sodium concentrations will change the luminal osmotic pressure and, hence, promote gut fluid secretions. Consequently, the elevated fluid volumes will alter gut motility as well as the consistency and size of the stool, a phenomenon which will further alter the gut behaviour and impact the local and systemic pharmacokinetics of drugs.

Development of a model that can predict the pharmacokinetics or pharmacodynamics of tenapanor is not straightforward due to its minimal systemic exposure and downstream alterations of gut behaviour as a result of inhibition of sodium absorption. Systemic PK could be used as a surrogate of the luminal concentrations to indirectly validate a model to link with a pharmacodynamic endpoint. However, a different strategy was employed, considering that tenapanor is a highly selective inhibitor of NHE3 and that NHE3 plays a major role in the absorption of sodium. This highlighted an opportunity to utilize a DDI modelling approach to shed light on the local luminal concentrations of unmeasured tenapanor. However, this modelling strategy is not a simple DDI simulation, particularly as induction of NHE3 occurs upon food digestion.

Several studies have shown how different food components regulate the recycling (intracellular endosome to the apical membrane) and function of NHE3. The translocation of NHE3 will dynamically change based on the food digestion process and transit of the chyme along the GI tract, leading to an increase in NHE3 function at the apical membrane [4,30]. Without including meal-induced NHE3 activity, the PBBM could not capture the food effect on the amount of sodium excreted in the stool. Changes in gastric emptying and gut transit times did not explain the reduction in stool sodium content when tenapanor was administered 30 min after a meal or several hours after (fasting).

The only constructive data available to explain this induction was from butyrate studies [13], butyrate consisting of a short chain fatty acid formed upon bacterial fermentation of undigested food in the colon. The study only reports NHE3 induction in the colon, but this modelling work suggests the increase occurs across the entire GI tract as the increased absorption only in the colon did not improve the predictive performance of the model. As tenapanor was administered 30 min or 1–3 h after a meal, sodium from food would be readily absorbed mainly in the small intestine within this time frame rather than within the colon. Therefore, NHE3 induction should be accounted for along the entire GI tract rather than only in the colon. Following usage of the butyrate induction kinetics and normalisation to sodium equivalents, the model captured the interplay between induction and inhibition of NHE3 as a function of time and regional concentrations of both sodium and tenapanor to accurately capture sodium PK. Although the assumption that sodium induces NHE3 in a similar manner to butyrate was a simplification of the in vivo conditions, it provides a compelling assumption to explain tenapanor mechanism of NHE3 inhibition relative to mealtime.

Despite these limitations and the challenges associated with the modelling of such complex mechanisms, PBBM was able to capture urine and stool sodium content within 1.25-fold error and predict the potential advantage of tenapanor formulation as an HCl salt vs. the free base, especially in the assessment of the long-term pharmacodynamics in patients with IBS-C.

A potential reason for the ability of the model to capture observed data within 1.25-fold error was the selectivity of tenapanor to inhibit NHE3, which plays a major role in the gastrointestinal absorption of sodium. Thus, applying a DDI modelling strategy was adequate to describe the clinical data focusing on the relationship between tenapanor and NHE3 impacting sodium PK. However, the modelling strategy might not be adequate in other cases where there are analytical challenges associated with the measurement of the biomarker or of internal standard secreted into the faeces. A future strategy could also evaluate the impact of disease state on NHE3 activity, including luminal acidity and mucus compromised integrity [31].

Alternative strategies might need to be implemented, e.g., co-administration of drugs with known PK where the regional luminal conditions in the PBBM can be optimised and validated based on the reference drug and then used to predict the regional luminal concentrations of the minimally absorbed drug. However, more studies are needed to define the criteria for which systemic drugs should be co-administrated to allow for realistic predictions of the luminal concentrations of the minimally absorbed co-dosed drug. For example, should the regional luminal concentrations of the reference drug be sensitive in terms of dissolution or absorption (or both), or should it be a non-sensitive drug? Should the systemic drug be co-administrated using formulations with different release profiles or a single release profile? For instance, a correlation was found between systemic mesalamine plasma PK and regional luminal concentrations in the GI tract [32]. Thus, mesalamine could be co-dosed and its PK data could be used to develop, optimise, and validate a PBBM that adequately predicts the relationship between regional luminal concentrations and plasma PK. After developing and verifying the PBBM for mesalamine, the corresponding luminal concentrations of a co-administered minimally absorbed drug could be predicted and potentially quantitatively correlated to PD/biomarker data.

However, a thorough evaluation is needed with regard to potential interactions between systemic probes with known behaviour being altered or sensitive to co-administered non-absorbed drugs. For example, co-administration of tenapanor with a systemically available probe drug that is sensitive to luminal conditions might not be appropriate, as the elevated sodium concentrations in the lumen could alter the behaviour of the systemic drug leading to a misinterpretation of the data. This could happen for a salt form of a probe systemic drug in which the counter ions might change the probe drugs solubility, as the luminal concentrations will be higher than the baseline levels. Another potential confounding situation would be for a probe systemic drug with regional absorption. Coadministration with tenapanor might change the PK of the probe drug, as tenapanor increases the motility of the GI tract. Thus, the systemic drug might be transported too quickly to the distal part of the GI tract, potentially affecting its absorption and altering its systemic PK.

## 5. Conclusions

A PBBM was developed to predict stool and urine sodium content in response to tenapanor administration in healthy subjects. Tenapanor is a minimally absorbed small-molecule compound that inhibits the sodium uptake transporter NHE3 and, thus, reduces the absorption of sodium from the GI tract. This particular modelling strategy was employed to gain confidence in the PBBM model of tenapanor by accurately predicting the food–drug interactions occurring in the GI tract between sodium and tenapanor.

The implemented modelling strategy, although simple, proved to be robust using the daily urine and stool sodium contents to validate the tenapanor PBBM model. A similar strategy could be used where an endogenous or exogenous biomarker can be tracked in the faeces, for example in the case of ileal bile acid transporter (IBAT) inhibitors, where bile acid absorption is blocked in the ileum and the extent of this action can be assessed by analysing the faecal bile acid content.

## Figures and Tables

**Table 1:** Sodium and tenapanor PBPK/PBBM model input parameters.

Parameter	Value (Unit)	
	Sodium (Na+)	Tenapanor HCl
**Physico-chemical properties**		
Molecular Weight	23	1217.97 [15]
log P	−10 ^a^	4.07 [15]
Compound Type	Neutral	Monoprotic base
pKa	-	6.6 (estimated with SIVA)
B/P	0.55	0.63
Fraction unbound in plasma, fu	1.0	0.001
**Absorption**		
Absorption model option	ADAM	
Formulation type	Solution	IR
Passive Permeability (10^−6^ cm/s)	0 (Caco2)	0.04 (MDCK) [3]
**Dissolution**		
Intrinsic solubility (mg/mL)	-	1.73 10^−4^ (estimated with SIVA)
CSR	-	10 (default)
PRC (h^−1^)	-	4 (default)
Salt limited solubility model	-	active
Drug solubility at pHmax (mg/mL)	-	8.267_@pH1.0_ [15] (free base)
K_sp_ value (mM^2^)	-	43.429 (back calculated from solubility at pHmax)
Solubility factor	-	17,328 _free base-(estimated with SIVA)_
Counterion		Hydrochloric acid
**Distribution model**	Mini-PBPK	
Distribution volume input type	Predicted	
Vss	0.495	0.370
Global tissue to plasma (Kp) Scalar	1	1
**Elimination model**		
Renal Clearance (L/h)	5.5 (%CV:30) (optimized) ^b^	-
Hepatic Clearance (L/h)		8.1 (%CV:30) ^c^
**Transport model**		
Gut NHE3 (Apical Influx)		
Jmax (pmol/min)	4900 (optimized) ^b^	-
Km (μM)	4700 [16] ^d^	-
**Regional relative abundance of NHE3** [17]		
Duodenum	0 ^e^	
Jejunum I–II	1 ^e^	
Ileum I–IV	1.5 ^e^	
Colon	0.4 ^e^	
**Transporter interaction—NHE3**		
Transporter	-	Apical influx (intestine)
Ki (μM)	-	0.005 [3]
**Food-stimulated NHE3 activity**		
Emax	5.72 ^f^	-
EC_50_ (μM)	1500 ^f^	-
γ	1.93 ^f^	

Note: ^a^ Lowest value allowed in Simcyp^®^; ^b^ parameter optimised to capture urine and stool excretion placebo data; ^c^ mass balance data [18]; ^d^ rat data; ^e^ the regional values were normalised with respect to jejunum 1 [17]; ^f^ Emax, EC_50_ (μM), and γ (Hill equation exponent) values were obtained from NHE3 induction studies with butyrate normalised to sodium equivalents [13]. This allowed the induction to be applied across the GI tract and not only in the colon (butyrate is produced by colonic microflora).

**Table 2:** Simulated and observed excretion of sodium in urine and stool after oral administration of tenapanor under fasted and fed conditions.

	Dose Schedule	Urine (mmol/Day)			Stool (mmol/Day)		
		Predicted	Observed	Pred/Obs	Predicted	Observed	Pred/Obs
Dose regimen study [2]	15 mg b.i.d	122.5	112.30	1.09	37.00	30.50	1.21
	30 mg b.i.d	123.00	114.90	1.07	38.4	27.00	1.42
	30 mg q.d	147.1	143.00	1.03	14.3	12.40	1.16
	30mg t.i.d	112.00	98.90	1.13	45.90	41.30	1.11
	60mg b.i.d	118.8	94.80	1.25	39.30	47.50	0.83
Food effect (15 mg b.i.d) [4]	Before meal				22.50	25.90	0.87
	After meal				19.70	17.20	1.14
	Fasting				15.00	14.10	1.07
Dose escalation study [3]	3 mg q.d				6.20	4.70	1.33
	10 mg q.d				9.00	9.50	0.95
	30 mg q.d				15.00	11.70	1.28
	100 mg q.d				14.70	15.40	0.95
AAFE		1.05			1.18		
AFE		1.11			1.10		

## Data Availability

The data presented in this study are available in this article and supplementary material.

## Supplementary Materials

The following supporting information can be downloaded at: https://www.mdpi.com/article/10.3390/pharmaceutics15122726/s1, Figure S1: Model drug free form experimental vs. predicted aqueous solubility of tenapanor using SIVA v4.; Figure S2: Predicted regional concentrations of sodium (linear scale) and tenapanor (log scale) using two scenarios.
