# Supplementary material for: Physiologically Based Biopharmaceutics Model (PBBM) of Minimally Absorbed Locally Acting Drugs in the Gastrointestinal Tract—Case Study: Tenapanor
Source: Pharmaceutics. 2023 Dec 4;15(12):2726. doi: 10.3390/pharmaceutics15122726 (PMC10747343; doi:10.3390/pharmaceutics15122726)
Supplement: Supplementary file 1 [file pharmaceutics-15-02726-s001.zip › pharmaceutics-2685138-supplementary.pdf]

pharmaceutics

MDPI

Supplementary Material: Physiologically Based Biopharmaceutics Model (PBBM) of Minimally Absorbed Locally Acting Drugs in the Gastrointestinal Tract—Case Study: Tenapanor

Konstantinos Stamatopoulos, Nena Mistry, Nikoletta Fotaki, David B. Turner and Brandon Swift

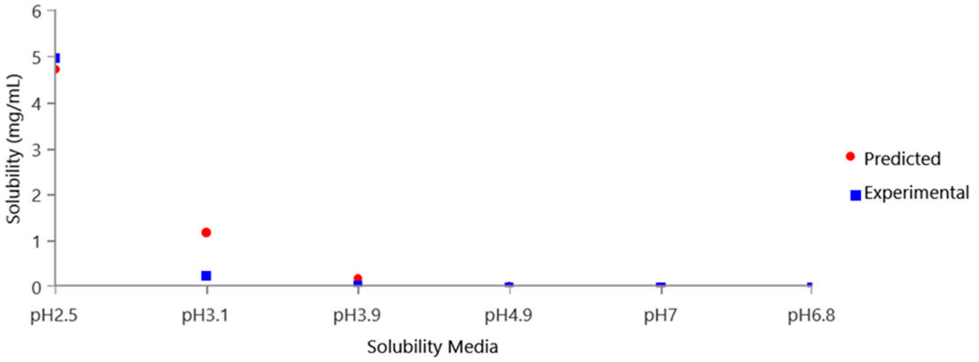

Figure S1. Model drug free form experimental vs. predicted aqueous solubility of tenapanor using SIVA v4.

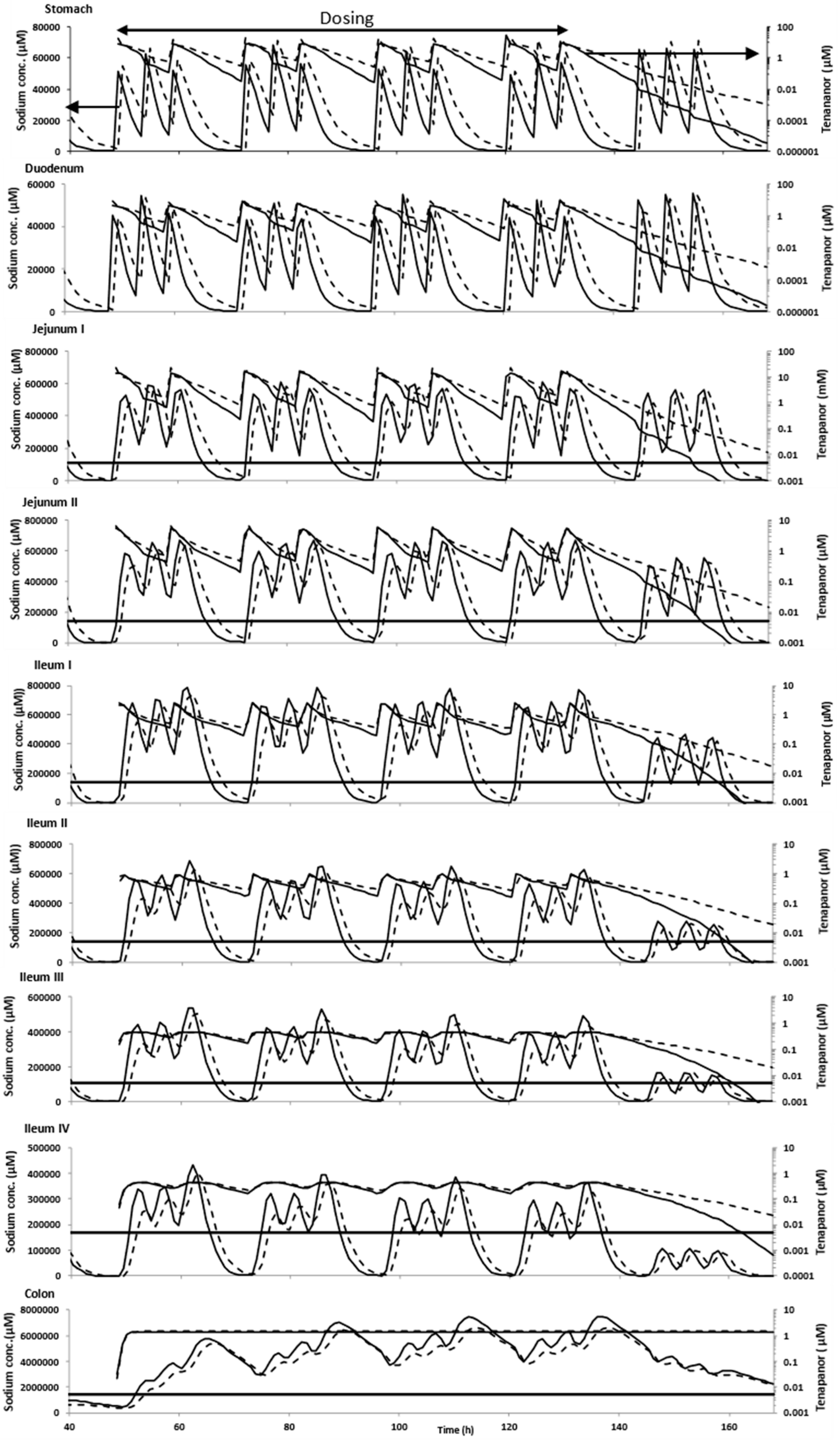

Figure S2. Predicted regional concentrations of sodium (linear scale) and tenapanor (log scale) using two scenarios; Gastric mean residence times of 1.35 h and tenapanor administration of 10 min before meal (—) and gastric mean residence times of 2.45 h and tenapanor administration of 60 min before

mean (- - -); the black solid horizontal line shows the target luminal concentration of tenapanor to reach IC_{50} (0.005 μM).
